# Is watchful waiting an alternative to adenotonsillectomy in children with mild to moderate obstructive sleep apnoea?

**DOI:** 10.1007/s00431-026-07330-6

**Published:** 2026-08-13

**Authors:** Stefan Kuhle, Christian F. Poets, Michael S. Urschitz

**Affiliations:** 1 Institute of Clinical Epidemiology, Public Health, Health Economics, Medical Statistics and Informatics, Medical University of Innsbruck, Innsbruck, Austria; 2 Department of Neonatology and Division of Paediatric Sleep Medicine, University Children’s Hospital, Tübingen, Germany; 3 Division of Paediatric Epidemiology, Institute of Medical Biostatistics, Epidemiology and Informatics, University Medical Centre of the Johannes Gutenberg-University, Mainz, Germany

**Keywords:** Apnoea, Adenotonsillectomy, Watchful waiting, Polysomnography, Sleep, Children

## Abstract

Adenotonsillectomy (ATE) has traditionally been the first-line treatment for obstructive sleep apnoea (OSA) in children. Increasing evidence suggests that many children with mild to moderate OSA may improve without surgery. This mini-review examines whether watchful waiting is a safe and effective alternative to ATE in otherwise healthy children with mild to moderate OSA. We searched PubMed from inception to January 2026 for randomised controlled trials (RCTs) comparing ATE with watchful waiting in children aged 1–16 years with OSA or sleep-disordered breathing confirmed by polysomnography or a validated symptom score. Two reviewers independently screened and extracted data. Results were analysed qualitatively. Five RCTs (seven published reports; *n* = 1287 children; mean age 6.1 years) met inclusion criteria. Spontaneous OSA resolution occurred in 44–55% of children managed with watchful waiting, compared with 57–79% following ATE. ATE produced statistically greater reductions in the apnoea–hypopnoea index in four of five trials, but between-group differences were modest. The largest and most consistent effects of ATE were seen for OSA symptom scores.

*Conclusion: *Watchful waiting is a reasonable option for young, nonobese children with mild OSA, given the relatively high rates of spontaneous resolution. ATE remains appropriate for children with moderate to severe OSA. Shared decision-making, individualised care and close follow-up are essential when watchful waiting is chosen.

**Table:** 

**What is Known:** • *Otherwise healthy children with obstructive sleep apnoea due to adenotonsillar hyperplasia are usually managed with adenotonsillectomy.* • *Young, non-obese, Caucasian children with more mild obstructive sleep apnoea, however, showed a surprisingly high probability of spontaneous resolution of the disease in one study.*
**What is New:** • *High rates of spontaneous resolution of obstructive sleep apnoea were observed in four independent studies, ranging from 44% to 55%.* • *Watchful waiting for several months may, thus, be a reasonable management option in a particular low-risk group of children with obstructive sleep apnoea.*

**Supplementary Information:**

The online version contains supplementary material available at https://doi.org/10.1007/s00431-026-07330-6.

## Introduction

Childhood obstructive sleep apnoea (OSA) is characterised by a cessation of airflow due to obstruction of the upper airway during sleep and occurs in 1 to 6% of children [1]. The condition is associated with significant short- and long-term complications and can negatively impact a child’s quality of life, behaviour and learning [1]. Nocturnal, attended, laboratory-based polysomnography (PSG) is considered the gold standard for diagnosing OSA [1]. PSG provides an objective, quantitative evaluation of disturbances in respiratory and sleep patterns, and an apnoea–hypopnoea index (AHI) or obstructive AHI (OAHI) > 1 or 2/h is commonly considered abnormal in children [1].

Early observational studies suggested that childhood OSA and, to some extent, also milder forms of nocturnal respiratory dysfunction (i.e., sleep-disordered breathing [SDB]) are associated with clinically significant neurobehavioural morbidity, including deficits in sustained and selective attention, alertness, emotion regulation, behaviour and scholastic performance [2]. Based on PSG reference values from healthy populations, there was broad agreement that snoring children with elevated OAHI should be treated to prevent or attenuate neurobehavioural morbidity.

The most common cause of OSA in otherwise healthy children without obesity is adenotonsillar hyperplasia, and immediate adenotonsillectomy (ATE) has traditionally been suggested as the primary treatment in these cases in the past. This recommendation, however, was based on the belief that OSA persists or aggravates over time and on the apparent benefits of ATE observed in uncontrolled before-after studies [1]. Since the landmark Childhood Adenotonsillectomy Trial (CHAT) in 2013 [3], several RCTs have examined ATE versus nonsurgical management strategies [4–7]. These studies, however, have not demonstrated a consistent, clinically decisive advantage of surgery across all outcome measures. Moreover, an unexpectedly large proportion of children in the control groups, particularly those with mild to moderate OSA, showed improvements in their OAHI without intervention.

These findings create a management dilemma for clinicians: they must weigh the likelihood of a spontaneous resolution against the potential benefits of a surgical procedure with its costs and documented risks. In this mini-review, we summarise the current evidence to examine whether watchful waiting is a safe and effective alternative to ATE in otherwise healthy children with mild to moderate OSA.

## Methods

We included RCTs that compared ATE with watchful waiting for the treatment of OSA or SDB in children aged 1 to 16 years. Participants had to have OSA confirmed by PSG or SDB identified by a validated symptom score or clinical evaluation (e.g., snoring, laboured breathing and sleep disruption).

A PubMed search (inception to January 2026) combined MeSH and free‑text terms for “child”, “obstructive sleep apnea”, “watchful waiting” and “tonsillectomy” (see Appendix). No language restrictions were applied.

Two reviewers independently screened titles and abstracts and then performed full‑text assessments of potentially eligible records. Study meta-data, participant characteristics, details of the intervention and control group, outcomes and type of analysis were extracted using a standardised electronic form. Any disagreements were resolved by third-party adjudication.

The primary outcome was resolution of OSA, defined as an OAHI < 1/h (or OAHI < 2/h [3]) on PSG. Secondary outcomes included AHI/OAHI and OSA/SDB symptom score. Results were analysed qualitatively only; no meta-analysis was performed.

## Results

The PubMed search returned 60 records; after title/abstract screening and full-text review, five RCTs (eight published studies) fulfilled the inclusion criteria [3–10]. Across the five trials, 1287 children (range 60–464) aged 2–12 years (mean 6.1 years) with OSA/SDB were enrolled.

Trials were initiated in the United States (*n* = 2), Europe (*n* = 1) and Australasia (*n* = 2) between 2008 and 2016 and published between 2013 and 2025. Median follow-up time at the primary outcome assessment was 9 months (range 6–12 months); one secondary study reported 36 months follow-up results.

Results for the primary outcome for each study are shown in Table 1. All studies reported PSG findings, but only four trials reported OSA resolution. In the watchful waiting arms, resolution rates were 44%, 46%, 50% and 55%; corresponding rates in the adenotonsillectomy arms were 57%, 60% and 79% (the POSTA trial did not report resolution in the surgical arm).

**Table 1:** Characteristics of included studies and resolution of obstructive sleep apnoea

Study	Study group	*N* (randomised)	*N* (treatment switching)	*N* (follow-up)	Follow-up time	Definition of resolution	Results and type of analysis
Au et al. 2021 [4]	*N* = 114, mean (range) age 8.4 years (6–11), 62% males, mild OSA (OAHI 1–5/h), single center in Hongkong/China	WW 57, ATE 57	WW 0, ATE 11	WW 36, ATE 35	9 months	OAHI < 1/h	WW 44%, ATE 57% (PP)
Fehrm et al. 2020 [5]	*N* = 60, mean (range) age 3.2 years (2–4), 57% males, mild to moderate OSA (OAHI 2- < 10/h), single center in Stockholm/Sweden (KATE trial)	WW 31, ATE 29	WW 1, ATE 1	WW 28, ATE 25	6 months	OAHI < 2/h	WW 50%, ATE 60% (PP)
Garetz et al. 2015 [8]	*N* = 453, mean (range) age 6.4 years (5–9.9), 48% males, OSA (OAHI 2–30/h), 6 sites in the US (CHAT trial)	WW 226, ATE 227	WW 16, ATE 8	WW 226, ATE 127	7 months	OAHI < 1/h	Not reported
Marcus et al. 2013 [3]	*N* = 464, mean (range) age 6.5 years (5–9.9), 49% males, OSA (OAHI 2–30/h), 7 sites in the US (CHAT trial)	WW 227, ATE 226	WW 16, ATE 16	WW 203, ATE 194	7 months	AHI < 2/h and OAHI < 1/h	WW 46%, ATE 79% (ITT)
Redline et al. 2023 [6]	*N* = 459, median (range) age 6 years (3–12.9), 50% males, habitual snoring and mild OSA (OAHI < 3/h), 7 centers in the US (PATS trial)	WW 227, ATE 231	WW 13, ATE 19	WW 227, ATE 231	12 months	OAHI < 1/h	Not reported
Sjölander et al. 2025 [9]	*N* = 60, mean (range) age 3.2 years (2–4), 57% males, mild to moderate OSA (OAHI 2- < 10/h), single center in Stockholm/Sweden (KATE trial)	WW 31, ATE 29	WW 13, ATE 4	WW 16, ATE 23	36 months	OAHI < 1/h	Not reported
Waters et al. 2020, 2021 [7, 10]	*N* = 190, mean (range) age 3.9 years (3–5), 54% males, PSQ score > 0.3 and no severe OSA (OAHI ≤ 10/h), 3 centers in Australia (POSTA trial)	WW 91, ATE 99	WW 4, ATE 1	WW 69, ATE 72	12 months	OAHI < 1/h	WW 55%*, ATE not reported (ITT)

*Among those with an OAHI > 1/h at baseline; results were reported in Waters et al. 2021

*ATE* adenotonsillectomy, *CI* confidence interval, *IQR* interquartile range, *ITT* intention to treat, *(O)AHI* (obstructive) apnoea/hypopnoea index, *OSAS* obstructive sleep apnoea syndrome, *PP* per protocol, *PSG* polysomnography, *PSQ* Pediatric Sleep Questionnaire, *RCT* randomised controlled trial, *WW* watchful waiting

Results for the secondary outcomes are shown in Table 2. The pre–post difference in the OAHI in the watchful waiting group at the primary follow-up assessment ranged from − 1.9 to 1.3. In four of the six studies that assessed the OAHI, the latter improved significantly following ATE compared to watchful waiting, although the absolute between-group differences were modest. The largest effect sizes in favour of ATE were seen for OSA symptom scores.

**Table 2:** Secondary outcomes

Study	Results WW group	Results ATE group
Au et al. 2021 [4]	Baseline OAHI (mean, SD): 2.4 (1.2) ∆OAHI (mean, SD): 0.3 (4.1) Baseline OSA-18 score (mean, SD): 56 (16) ∆OSA-18 score (mean, SD): −3.6 (14.1)	Baseline OAHI (mean, SD): 2.7 (1.1) ∆OAHI (mean, SD): − 1.4 (2.0) Baseline OSA-18 score (mean, SD): 63 (17) ∆OSA-18 score (mean, SD): − 17.3 (19.7)
Fehrm et al. 2020 [5]	Baseline OAHI (mean, SD): 5.0 (2.2) ∆OAHI (mean, 95%CI): − 1.9 (− 3.0, − 0.9) Baseline OSA-18 score (median, IQR): 59 (48, 69) ∆OSA-18 score (mean, 95% CI): − 4.5 (− 12, 1.5)	Baseline OAHI (mean, SD): 4.9 (1.9) ∆OAHI (mean, SD): − 2.9 (− 4.0, − 1.9) Baseline OSA-18 score (median, IQR): 59 (49, 74) ∆OSA-18 score (mean, 95% CI): − 23.5 (− 31.5, − 15)
Garetz et al. 2015 [8]	Baseline AHI (mean, SD): 6.7 (0.4) ∆AHI: not reported Baseline PSQ-SRBD score (mean, SD): 0.5 (0.2) ∆PSQ-SRBD score (mean, SD): 0.0 (0.2) Baseline OSA-18 score (mean, SD): 54.1 (18.8) ∆OSA-18 score (mean, SD): − 4.5 (19.3)	Baseline AHI (mean, SD): 6.9 (0.4) ∆AHI: not reported Baseline PSQ-SRBD score (mean, SD): 0.5 (0.2) ∆PSQ-SRBD score (mean, SD): − 0.3 (0.2) Baseline OSA-18 score (mean, SD): 53.1 (18.3) ∆OSA-18 score (mean, SD): − v21 (16.5)
Marcus et al. 2013 [3]	Baseline AHI (median, IQR): 4.5 (2.5, 8.9) ∆AHI (median, IQR): − 1.6 (− 3.7, 0.5) Baseline PSQ-SRBD score (mean, SD): 0.5 (0.2) ∆PSQ-SRBD score (mean, SD): 0.0 (0.2)	Baseline AHI (median, IQR): 4.8 (2.7, 8.8) ∆AHI (median, IQR): − 3.5 (− 7.1, − 1.8) Baseline PSQ-SRBD score (mean, SD): 0.5 (0.2) ∆PSQ-SRBD score (mean, SD): − 0.3 (0.2)
Redline et al. 2023 [6]	Baseline AHI (median, IQR): 0.6 (0.3, 1.2) ∆AHI (median, IQR): 0.1 (− 0.3, 0.9) Baseline PSQ-SRBD score (mean, SD): 0.5 (0.2) ∆PSQ-SRBD score (mean, SD): − 0.1 (0.2) Baseline OSA-18 score (mean, SD): 52.7 (17.4) ∆OSA-18 score (mean, SD): − 6.0 (14.6)	Baseline AHI (median, IQR): 0.5 (0.1, 1.1) ∆AHI (median, IQR): ATE − 0.2 (− 0.6, 0.2) Baseline PSQ-SRBD score (mean, SD): 0.5 (0.2) ∆PSQ-SRBD score (mean, SD): − 0.2 (0.2) Baseline OSA-18 score (mean, SD): 51.2 (15.7) ∆OSA-18 score (mean, SD): − 15.8 (14.4)
Sjölander et al. 2025 [9]	Baseline OAHI (mean, SD): 5.0 (2.2) ∆OAHI (mean, 95%CI): − 3.6 (− 4.5, − 2.6) Baseline OSA-18 score (median, IQR): 58.5 (48, 69) ∆OSA-18 score (mean, 95%CI): − 13.8 (− 25.0, − 2.5)	Baseline OAHI (mean, SD): 4.9 (1.9) ∆OAHI (mean, 95%CI): − 3.7 (− 4.8, − 2.6) Baseline OSA-18 score (median, IQR): 59 (49, 74) ∆OSA-18 score (mean, 95%CI): − 26 (− 37.7, − 14.4)
Waters et al. 2020, 2021 [7, 10]	Baseline OAHI (mean, SD): 1.9 (2.0) OAHI at follow-up (mean, SD): 1.3 (2.0) Baseline PSQ score (mean, SD): 0.60 (0.16) PSQ score at follow-up (mean, SD): 0.53 (0.21)	Baseline OAHI (mean, SD): 1.9 (1.9) OAHI at follow-up (mean, SD): 0.3 (0.5) Baseline PSQ score (mean, SD): 0.62 (0.15) PSQ score at follow-up (mean, SD): 0.24 (0.18)

*ATE* adenotonsillectomy, *CI* confidence interval, *IQR* interquartile range, (*O)AHI* (obstructive) apnoea/hypopnoea index, *OSA* obstructive sleep apnoea, *PSG* polysomnography, *PSQ* Pediatric Sleep Questionnaire, *RCT* randomised controlled trial, *SD* standard deviation, *SRBD* sleep-related breathing disorder, *WW* watchful waiting

## Discussion

Four studies examined resolution of OSA as an outcome and demonstrated high rates of spontaneous OSA resolution in the watchful waiting group, ranging from 44 to 55%. The estimates, however, may be biased by treatment switching and lost-to-follow-up, affecting 0 to 9% (for treatment switching) and 6 to 37% (for lost-to-follow-up) of patients. Estimates may be further affected by the type of analysis; two studies reported results of an intention-to-treat analysis. While treatment switching was low, the higher numbers of lost-to-follow-up prevented a clear conclusion of the review.

Four RCTs showed a statistically significant improvement in AHI or OAHI following ATE compared to watchful waiting, but the group difference in change from baseline was often small and likely of limited clinical significance. The largest effect sizes in these trials were seen for symptom scores, but the magnitude of the effect varied across subgroups. For example, the CHAT trial [3, 8] reported smaller improvements in African-American children, and the Karolinska Adenotonsillectomy (KATE) trial [5] found that children with mild OSA benefited less from ATE than those with moderate OSA.

Predictors for a spontaneous resolution of OSA under watchful waiting were assessed in a subgroup of children from the CHAT trial. Among 194 participants, 42% experienced spontaneous resolution of OSA at the 7-month follow-up. A low baseline OAHI and a normal waist circumference were associated with OSA resolution [11]. In children with a baseline OAHI between 2.6/h and 4.6/h, approximately 50% experienced spontaneous resolution on PSG.

Watchful waiting is noninvasive and involves monitoring the condition over time to determine if it resolves or worsens, requiring other interventions. In children with OSA, it avoids surgical risks such as postoperative pain, haemorrhage and anaesthesia complications [1]. Watchful waiting may also be more cost-effective than ATE, potentially reducing healthcare costs by decreasing the need for surgical procedures and hospital stays. However, this approach was not evaluated before 2013, when it became the control intervention in the first RCT on the efficacy of ATE in children with OSA [12]. Since then, watchful waiting has been used as the control intervention in five RCTs [3–7].

Despite its potential benefits, watchful waiting has some drawbacks. First, it is not a well-standardised intervention, as it may include several supportive care measures such as nasal saline rinses or anti-inflammatory agents. Second, one concern is the possibility of disease progression or worsening of symptoms during the observation period. Most studies have found that only a small percentage of children with mild OSA experience significant worsening of their condition over time. For example, in one study, 14% of patients had relevant disease progression under watchful waiting, compared to 6% following ATE [4]. Delaying treatment could lead to persistent neurocognitive or behavioural impairments; however, ATE did not show significant improvements in neurocognitive function [3, 4]. Additionally, a study comparing early ATE to ATE after 12 months of watchful waiting found no benefits for intellectual ability [10]. Third, monitoring children under watchful waiting can be challenging. It is difficult to predict which cases of mild to moderate OSA will resolve and which will worsen, requiring close follow-up to identify those who need surgery, since the natural course of OSA is generally not well understood. Spontaneous improvements may occur due to atrophy of tonsillar tissue, pharyngeal growth, night-to-night variability or regression to the mean. Fourth, improvements in PSG parameters do not always correlate with symptom improvement or quality of life. Children may still experience symptoms despite an improvement (or resolution) in their OAHI. One study found that only 15% of children with significant symptoms at baseline in the watchful waiting group experienced meaningful improvement in or resolution of their OSA-related symptoms based on parental questionnaires [11]. Therefore, close and comprehensive monitoring of both PSG parameters and symptoms is necessary to ensure that children do not experience adverse outcomes due to delayed intervention.

There are notable gaps in the evidence regarding the management of mild to moderate OSA in children. For example, age may be important for both spontaneous resolution of symptoms and neurobehavioural morbidity, which would affect management. Beyond baseline OAHI and normal waist circumference, little is known about predictors of spontaneous resolution of OSA. Also, tonsil size is a potential predictor of OSA that was not systematically studied in the studies contributing to this review. Future research should identify predictors of spontaneous resolution in children with OSA. Artificial intelligence offers a promising approach to better characterise OSA phenotypes in children. Studies measuring long-term, patient-centred outcomes, such as quality of life, neurocognitive development and behaviour, rather than focusing solely on short-term symptom relief or PSG parameters, are also needed. While improvements in PSG parameters are important, they do not correlate well with symptom improvement or quality of life, highlighting the need for consideration of other clinical factors. Lastly, RCTs with longer follow-up periods are needed to better understand the long-term effects of ATE and watchful waiting. This would allow for the assessment of both the benefits and risks of each approach and help identify which patients benefit most from each intervention.

Shared decision-making with families is essential in managing mild to moderate OSA in children. This approach involves discussing the risks, benefits and uncertainties of both watchful waiting and ATE with caregivers. ATE may provide rapid symptom relief and improve sleep quality but carries surgical risks, including pain, haemorrhage and respiratory complications. Watchful waiting avoids these risks but may lead to disease progression or delayed treatment. Spontaneous resolution can occur in some cases, and the degree of improvement may vary, allowing some parents to choose less invasive options. Treatment plans should be individualised, considering the child’s symptoms, history, family preferences and co-existing conditions like obesity. Adjunctive therapies, such as lifestyle changes (e.g., weight loss for children with obesity) or medical treatments (e.g., nasal corticosteroids or leukotriene receptor antagonists [13]) should also be considered as part of a comprehensive management plan.

## Limitations

Due to the limited scope of this mini-review, we focused on PubMed, PSG indices and symptom scores. While the OSA-18 addresses health-related quality of life, other measures important to parents, such as alertness or attention, were not addressed.

Variability in study settings, PSG analyses, diagnostic criteria, OSA severity, watchful waiting protocols, outcome definitions and analysis strategies (ITT vs. PP) precluded pooling of the results and performing a meta-analysis. For instance, studies used different indices (AHI vs. OAHI) and thresholds (OAHI > 1/h vs. > 2/h) to define OSA, its severity and resolution.

Consequently, our findings should be interpreted as indicative trends rather than precise estimates applicable to all clinical settings.

## Conclusions

Young, nonobese, Caucasian children with mild OSA may be managed with watchful waiting and regular follow-up, given the high likelihood of spontaneous resolution. More research is needed to establish clear guidelines for managing mild to moderate OSA in children, with a focus on standardised outcome measures and identifying predictors of success with watchful waiting.

## Supplementary Information

Below is the link to the electronic supplementary material.

ESM 1 (DOCX 17.5 KB)

## Data Availability

No datasets were generated or analysed during the current study.
