# Supplementary material for: Is watchful waiting an alternative to adenotonsillectomy in children with mild to moderate obstructive sleep apnoea?
Source: Eur J Pediatr. 2026 Aug 13;185(9):663. doi: 10.1007/s00431-026-07330-6 (PMC13473323; doi:10.1007/s00431-026-07330-6)
Supplement: Supplementary file 1 [file 431_2026_7330_MOESM1_ESM.docx]

**Appendix: Search terms and strategy.**

(

("Child"[Mesh] OR "Pediatrics"[Mesh] OR

child[tiab] OR children[tiab] OR pediatric[tiab] OR paediatric[tiab] OR

adolescen*[tiab] OR teen[tiab] OR youngster[tiab])

)

AND

(

"Sleep Apnea, Obstructive"[Mesh] OR

("obstructive sleep apnea"[tiab] OR "OSA"[tiab] OR

"obstructive sleep apnoea"[tiab] OR "sleep‑disordered breathing"[tiab] OR

"sleep disordered breathing"[tiab] OR "sleep‑related breathing disorder"[tiab])

)

AND

(

"Watchful Waiting"[Mesh] OR

(watchful wait*[tiab] OR expectant management[tiab] OR

expectant[tiab] OR conservative management[tiab] OR

conservative[tiab] OR "active surveillance"[tiab] OR

"non‑surgical"[tiab] OR "non surgical"[tiab] OR

"observation"[tiab] OR "observational"[tiab])

)

AND

(

"Adenoidectomy"[Mesh] OR "Tonsillectomy"[Mesh] OR

(adenoidectom*[tiab] OR tonsillectom*[tiab] OR

"adenotonsillectomy"[tiab] OR "adenotonsillectom*"[tiab] OR

"tonsil removal"[tiab] OR "adenoid removal"[tiab])

)

AND

(

"Randomized Controlled Trial"[Publication Type] OR

(randomi?ed controlled trial[tiab] OR

rct[tiab] OR "randomised controlled trial"[tiab] OR

"controlled clinical trial"[tiab] OR

"clinical trial"[tiab])

)
